# Uptake and acceptability of oral HIV self-testing in the context of assisted partner services in Western Kenya: A mixed-methods analysis

**DOI:** 10.1371/journal.pgph.0003960

**Published:** 2024-11-15

**Authors:** Victor Mudhune, Unmesha Roy Paladhi, Mercy Owuor, Kenneth Ngure, David A. Katz, George Otieno, Monisha Sharma, Sarah Masyuko, Edward Kariithi, Carey Farquhar, Rose Bosire

**Affiliations:** 1 Centre for Global Health Research, Kenya Medical Research Institute, Kisumu, Kenya; 2 School of Public Health, Jomo Kenyatta University of Agriculture and Technology, Nairobi, Kenya; 3 Department of Global Health, University of Washington, Seattle, Washington, United States of America; 4 Department of Epidemiology, University of Washington, Seattle, Washington, United States of America; 5 Independent Qualitative Researcher, Nairobi, Kenya; 6 PATH-Kenya, Kisumu, Kenya; 7 Departments of Epidemiology and Medicine, University of Washington, Seattle, Washington, United States of America; 8 Centre for Clinical Research, Kenya Medical Research Institute, Nairobi, Kenya; The Chinese University of Hong Kong, HONG KONG

## Abstract

Integrating HIV self-testing (HIVST) into assisted partner services (APS) has potential to increase identification of people with HIV in the community, but little is known about acceptability of HIVST among partners traced via APS. We assessed characteristics of APS partners testing with HIVST, and factors influencing HIVST uptake and acceptability in a cluster-randomized control trial on APS+HIVST. Using convergent parallel mixed-methods design, we evaluated socio-demographic and behavioral characteristics of APS partners who were offered HIVST or provider-delivered testing, and purposively selected a sub-set of partners for in-depth interviews (IDIs). Descriptive and log-binomial regression analyses were performed controlling for health facility clusters, while IDIs were thematically analyzed applying the theoretical framework of acceptability. Among 3312 partners who were offered HIVST or provider-administered testing through APS, 2724 (82.2%) used HIVST. There was no association between partner demographics and HIVST uptake. HIVST use was less likely than provider-delivered testing among those identified as a casual (adjusted relative risk (aRR) = 0.93; 95% Confidence Interval (CI) 0.88–0.98) or transactional (aRR = 0.90; 95% CI 0.87–0.94) partner compared to those in a defined relationship. HIVST use was slightly lower among those offered the option of an additional kit when compared to those only offered one kit (aRR = 0.93; 95% CI 0.88–0.98). In the IDIs (N = 24), partners reported that HIVST was a viable option for individuals who do not find provider-delivered testing suitable or convenient. For the APS partners, ‘intervention coherence’, ‘self-efficacy’, and ‘ethicality’ presented as most significant theoretical framework of acceptability constructs. APS providers played a critical role in creating HIVST awareness and driving acceptability. Increasing HIVST awareness and providing tailored solutions will empower APS clients optimize their HIV testing decisions. Providers should consider context of the partner’s sexual encounter and extend counselling support when recommending HIVST within APS.

## Introduction

Globally an estimated 86% of people living with HIV (PLWH) knew their status as at the end of 2022 [1], with a significant testing gap for populations with low access to HIV testing services (HTS) which limits progress towards the UNAIDS 95-95-95 goals [2]. In sub-Saharan Africa (SSA), knowledge of HIV status has steadily increased to 84% in 2020 [3], however regional, sex and age disparities persist. Community acceptance of integrated HIV testing strategies offers an opportunity to reach specific population segments and increase diagnosis of HIV.

Assisted partner services (APS), a public health strategy to test the sexual and drug-injecting partners of PLWH, has been shown to increase testing uptake and yield [4, 5], to be cost-effective [6] and does not increase intimate partner violence [5, 7]. In 2016, the World Health Organization (WHO) recommended incorporation of APS into routine HTS [8], however there have been challenges in implementing APS at scale especially in SSA [9]. Kenya was among the first African countries to develop guidelines on APS implementation, with a focus on referring partners to health facilities for HIV testing. However, there are important challenges to facility-based testing, including low partner turnout in the context of APS [10, 11]. APS partners referred to testing but not showing up at health facilities are more likely to be males, non-main partners, partners of short relationship duration and those aged <25 years [12]. Other reasons behind low provider-delivered facility-based testing include lack of trust in HTS providers [7], and stigma associated with healthcare facilities [13].

Oral HIV self-testing (HIVST) is an acceptable testing alternative for populations with HIV risk indication [14, 15], is accurately performed by the majority of users [16], and is preferred by some because of its privacy and confidentiality [17]. However, there are concerns around lack of counseling, possible user error, test inaccuracy and inadequate linkage to care [17, 18]. Individual characteristics [19, 20], in addition to country setting and type of approach [21], have been shown to affect HIVST acceptability with variability in uptake between 22% to 94% [17, 19]. Successful HIVST distribution strategies include via sexual partners at antenatal clinics [22], door-to-door [23], transactional sex settings [24], and community dispensing pharmacies [25]. Incorporation of HIVST into APS may address challenges associated with facility-based testing. To be useful in this manner, HIVST needs to be acceptable to partners and should not come with increased risk of social harm.

Prior studies have found that HIVST distribution to partners of women attending antenatal clinics increases testing and treatment initiation among sexual partners [22, 26], with no reported factors associated with partner testing [27]. While the women considered HIVST highly acceptable and confidential [28], they expressed concerns about lack of support to ensure linkage to care, uncertainty about partner reaction and potential for intimate partner violence [29]. Understanding drivers of HIV testing preferences outside of antenatal clinics, specifically in implementation APS settings, is important to determine potential impact of combined intervention at scale.

Within a cluster-randomized controlled trial that evaluated the effectiveness of offering partners of index APS clients the option of HIVST vs. standard provider-delivered HIV testing (APS-HIVST Study) [30], we assessed the acceptability of HIVST, and evaluated individual characteristics associated with partner uptake of HIVST within APS in Western Kenya. This study adds on to the growing body of evidence on HIVST in SSA and provides a novel perspective to examine whether HIVST can be successfully integrated into APS.

## Methods

### Study design

We conducted a convergent parallel mixed-methods study with joint interpretation of cross-sectional data from participants enrolled in a study on oral HIVST integration into APS, and subsequent in-depth interviews (IDIs) with selected participants after experiencing study intervention. The parent study was a cluster randomized controlled trial conducted at 24 health facilities in Kisumu and Homa Bay Counties in Western Kenya [30]. Facilities were stratified by HIV testing volume (average number of HIV tests performed at the site), and APS performance during the initial study period, and then randomized 1:1 into the intervention arm, where partners of index clients were offered the option to receive provider-delivered testing or HIVST (APS+HIVST); or the control arm, where partners were offered provider-delivered HIV testing only (Standard APS). This analysis focuses on participants enrolled at intervention sites (APS+HIVST) only, an arm where both HIVST and provider-delivered testing was offered based on client preference.

### Study site and setting

Recruitment and HIV testing was conducted between 11^th^ March 2021 and 31^st^ July 2022, and leveraged on existing integrated HIV prevention, care and treatment programs supported by President’s Emergency Plan for AIDS Relief (PEPFAR) at Ministry of Health facilities in Western Kenya. Data for this analysis were collected from 12 public health facilities equally distributed between Kisumu and Homa Bay counties: two regions with the highest HIV prevalence in Kenya at 17.5% and 19.6% respectively [31]. The facilities provided HIV testing and comprehensive care for PLWH. Outside of the study, oral HIVST is occasionally provided free-of-charge at government health facilities and is available for purchase at local community pharmacies.

### Study procedures

The study protocol has been previously published [32]. Briefly, sexual partner contact information was elicited from consenting index clients who were diagnosed with HIV at participating facilities. Inclusion criteria for partners were aged ≥18 years old, and willing to participate in the study. Partners were excluded if they were pregnant or reported intimate partner violence (IPV) during the previous month. Partners were confidentially contacted by phone and, if unsuccessful via phone or if no phone number was available, physical home visits and notified of their potential HIV exposure.

At APS+HIVST intervention sites, participants were provided with the option to either collect an HIVST kits(s) at designated community pharmacies and communicate the test results to HTS providers or receive provider-administered HIV testing within the community or at a health facility (standard APS). For the first 8 months of the study, participants were offered one HIVST kit, and for the next 8 months, they were offered an option of a second kit if they desired. All study HIV tests were provided free of charge. Participants who reported a positive test result using HIVST were asked to confirm their results with facility-based testing. All participants with confirmed HIV diagnoses were offered APS for their sexual partners and linked to HIV care.

IDIs were conducted with a sub-set of study participants at intervention (APS+HIVST) sites who were willing to share their experience. A purposive sampling approach was used to select 24 sexual partners across the 12 intervention facilities weighted by number of participants enrolled, ensuring equal representation by gender, self-selected HIV testing method, HIV outcome status, and county location of health facilities. The IDIs were conducted in April 2022.

### Data collection

#### Quantitative data

At enrollment, partner demographic characteristics were collected using interviewer-administered survey on open-source Open Data Kit platform [33], administered through tablets. The survey included data on HIV testing history, sexual behavior in the last 12 months, substance use, and definition of sexual relationship to the identified partners. The HIV testing method of choice was captured by the HTS providers.

#### Qualitative data

The IDIs were conducted using a semi-structured interview guide, which included questions on HIV testing knowledge and experience before APS, testing method decision drivers, HIV testing experience during APS, APS and HIVST delivery, and recommendations for integrating HIVST into APS. The IDI guide was piloted using sample participants and refined by study team (VM, MO, URP, RB) before actual interviews. Interviews were conducted in either English, Swahili, or Dholuo based on participant’s language preference, by a trained and experienced qualitative interviewer who was not part of the APS implementation team. The study team (VM, MO, RB) debriefed periodically to evaluate saturation and arising concerns. All interviews were conducted at the health facilities, audio-recorded, transcribed verbatim, and translated to English before analysis.

### Data analysis

#### Quantitative data

Using baseline data for partners from the 12 APS+HIVST intervention sites, descriptive statistics were summarized for demographics, sexual behavior, number of HIVST offered and HIV testing history. HIV risk score categorization based on HIV incidence (high, medium or low) was calculated from respondent’s age, occupation, marital status, number of sexual partners in the last 12 months, and HIV testing history, with weighted scores obtained from a risk-score algorithm developed and validated within the local community [34]. We performed log-binomial regression analysis with participant’s demographic characteristics, sexual behavior, testing history, number of HIVST kits offered, and risk categorization as exposure variables, and HIV testing method as the outcome, adjusting for clustering at the health facilities. We performed chi-square test for independence using Cramer’s V as an effect size measure, with cut-offs interpreted based on degrees of freedom [35]. Selection of variables for the final multivariate generalized linear model using backward elimination procedure was guided by overall significance test using a 0.2 cutoff, while adjusting the standard error for clustering at the health facilities. The final statistical tests were interpreted at 0.05 level of significance. Analysis was conducted using STATA (Release 17, 2021. StataCorp LLC, College Station, TX).

#### Qualitative data

Inductive coding was performed on five IDI transcripts by two independent coders, grouping the codes by constructs defined in the theoretical framework of acceptability [36], while also generating insights. The coded scripts were discussed to evaluate application of code, with further refinement of the code categories and definitions to develop the codebook. The codebook was applied to the rest of the transcripts independently by the two coders, developing additional codes based on findings. The first author iteratively ensured that all relevant text had been consistently coded; calculated Krippendorff’s c-Alpha-binary for inter-coder agreement based on mutually coded scripts (α = 0.631) and reconciled any disagreements [37]. The findings were synthesized and shared with the independent coders for validation. We present findings using the theoretical framework of acceptability [36]. Data analysis was conducted using ATLAS.ti Web (Version 3.15.0-2022-03-09).

### Ethical considerations

Ethical approval was obtained from the Kenyatta National Hospital—University of Nairobi Ethics and Research Committee (P465/052017) and University of Washington Institutional Review Board (STUDY000002420). No reimbursement was provided to participants for participation in the main study, however reimbursement for time and cost was made for interview respondents. Written informed consent was obtained from study participants before enrolling into APS, and verbal consent was obtained before referral for HIVST kits. All participants provided additional written informed consent for IDI participation.

## Results

### Quantitative analysis

Of the 3,312 partners enrolled in APS included in the analysis, 69.3% presented to health facilities in Homa Bay County, majority (60%) were male, 54.1% were self-employed, 81.1% were monogamously married or cohabiting, and the median age was 35 years (Table 1). Most partners were offered one HIVST kit (63.7%), had previously tested for HIV (96.2%), and intended to test for HIV individually instead of via couple testing (99.5%). The index client who named the partner was most commonly husband/wife or boyfriend/girlfriend (64.7%), with some casual partners (30.9%), transaction partners (4.3%) and one reported case of coerced sexual encounter. Only 1.4% identified as members of a key population, and 62.5% were classified as being at low risk of acquiring HIV based on the HIV risk score. Most partners opted to test using HIVST (82.2%).

**Table 1:** Characteristics of partners receiving assisted partner services, stratified by HIV test preference.

	Total N = 3312	Provider-Delivered Test n = 588 (17.8%)	HIV Self-Testing n = 2724 (82.2%)
	n (%) or median (IQR)	n (%) or median (IQR)	n (%) or median (IQR)
**Age (in years)**	35 (31–41)	35 (31–41)	35 (31–41)
**Gender**			
Female	1325 (40.0)	224 (16.9)	1101 (83.1)
Male	1987 (60.0)	364 (18.3)	1623 (81.7)
**County**			
Kisumu (Urban)	1018 (30.7)	192 (18.9)	826 (81.1)
Homa Bay (Rural)	2294 (69.3)	396 (17.3)	1898 (82.7)
**Health Facility**			
Nyangiela SCH	277 (8.4)	37 (13.4)	240 (86.6)
Kabondo SCH	387 (11.7)	54 (14.0)	333 (86.0)
Rachuonyo DH	928 (28.0)	215 (23.2)	713 (76.8)
Othoro SCH	249 (7.5)	42 (16.9)	207 (83.1)
Kowino Dispensary	158 (4.8)	13 (8.2)	145 (91.8)
Simba Opepo Dispensary	121 (3.7)	38 (31.4)	83 (68.6)
St. Elizabeth Chiga Hospital	381 (11.5)	37 (9.7)	344 (90.3)
Airport H.C.	84 (2.5)	30 (35.7)	54 (64.3)
Ojola H.C.	111 (3.4)	33 (29.7)	78 (70.3)
Port Florence Hospital	163 (4.9)	41 (25.2)	122 (74.8)
Tala Dispensary	276 (8.3)	29 (10.5)	247 (89.5)
Kauma H.C.	177 (5.3)	19 (10.7)	158 (89.3)
**Marital status**			
Currently Single^a^	503 (15.2)	85 (16.9)	418 (83.1)
Married/Cohabiting Monogamous	2686 (81.1)	475 (17.7)	2211 (82.3)
Married Polygamous	123 (3.7)	28 (22.8)	95 (77.2)
**Education Level**			
Never attended school/Some Primary School	297 (9.0)	44 (14.8)	253 (85.2)
Completed Primary School	1305 (39.4)	226 (17.3)	1079 (82.7)
Completed Secondary School	1181 (35.7)	242 (20.5)	939 (79.5)
Attended Tertiary Institution	529 (16.0)	76 (14.4)	453 (85.6)
**Occupation**			
Formal Employment^b^	234 (7.1)	44 (18.8)	190 (81.2)
Informal Employment^c^	730 (22.1)	124 (17.0)	606 (83.0)
Self-employed	1786 (54.1)	309 (17.3)	1477 (82.7)
Unemployed	424 (12.8)	94 (22.2)	330 (77.8)
Student	125 (3.8)	14 (11.2)	111 (88.8)
**Monthly Household Income** ^**d**^			
< 10,000	2310 (69.8)	426 (18.4)	1884 (81.6)
10,000–50,000	980 (29.6)	155 (15.8)	825 (84.2)
> 50,000	22 (0.7)	7 (31.8)	15 (68.2)
**Key Population** ^**e**^		** **	
Yes	45 (1.4)	7 (15.6)	38 (84.4)
No	3267 (98.6)	581 (17.8)	2686 (82.2)
**Number of HIVST Kits Offered**			
One Kit	2109 (63.7)	324 (15.4)	1785 (84.6)
Two Kits	1203 (36.3)	264 (21.9)	939 (78.1)
**Ever Tested for HIV Before**			
Yes	3186 (96.2)	571 (17.9)	2615 (82.1)
No	126 (3.8)	17 (13.5)	109 (86.5)
**Current Testing Intent**			
Individual	3296 (99.5)	581 (17.6)	2715 (82.4)
Couple	16 (0.5)	7 (43.8)	9 (56.3)
**Sexual Relationship with Index Case**			
Relationship Sex^f^	2144 (64.7)	329 (15.3)	1816 (84.7)
Casual Sex	1023 (30.9)	223 (21.8)	800 (78.2)
Transactional Sex	144 (4.3)	36 (25.0)	108 (75.0)
Coerced Sex	1 (0.03)	0 (0.0)	1 (100.0)
**HIV Risk Score Categorization** ^**g**^			
Low Risk	2071 (62.5)	353 (17.0)	1718 (83.0)
Medium Risk	783 (23.6)	139 (17.8)	644 (82.3)
High Risk	458 (13.8)	96 (21.0)	362 (79.0)

^a^ Currently single include never married, single, divorced, separated, widow or widower.

^b^ Formal employment include trade, sales or service occupation.

^c^ Informal employment include manual occupation.

^d^ Average currency exchange rate over the data collection period: 1 USD = KSh. 110.60.

^e^ Key population include PWID, AGYW, FF, FSW, MSM.

^f^ Relationship saex include Wife/Husband and Boyfriend/Girlfriend.

^g^ Risk scoring based on sociodemographic and behavioral characteristics: Muttai et al, 2021.

In univariate analysis, students were more likely to test using HIVST compared to those who were unemployed (Relative Risk (RR) = 1.14, 95% Confidence Interval (CI) 1.04–1.26), though this association was not statistically significant in the multivariate model (Table 2). In the multivariate model, adjusting for clustering at health facilities, HIVST use was slightly less likely among those offered two kits when compared to those offered one kit (aRR = 0.93, 95% CI 0.88–0.98). Additionally, HIVST use was less likely among partners named by casual (RR = 0.93, 95% CI 0.88–0.98) or transactional (RR = 0.90, 95% CI 0.87–0.94) partner, compared to those named by a spouse or other person they were in a relationship with.

**Table 2:** Characteristics associated with partner preference for HIV self-testing within assisted partner services.

	Relative Risk (RR)^a^		Adjusted RR^a^	
Variable	RR (95% CI)	p-value	RR (95% CI)	p-value
**Age**		** **		
≤29	Ref	Ref		
30–39	0.99 (0.96–1.02)	0.430		
40–49	0.99 (0.94–1.04)	0.634		
≥50	0.97 (0.91–1.04)	0.429		
**Gender**		** **		
Male	Ref	Ref		
Female	0.98 (0.94–1.02)	0.404		
**County**				
Kisumu (Urban)	Ref	Ref		
Homa Bay (Rural)	1.02 (0.89–1.17)	0.774		
**Marital status**				
Currently Single	Ref	Ref		
Married/Cohabiting Monogamous	0.99 (0.96–1.02)	0.537		
Married Polygamous	0.93 (0.84–1.03)	0.178		
**Education Level**				
Never attended school/Some Primary school	Ref	Ref	Ref	Ref
Completed Primary school	0.97 (0.92–1.03)	0.291	0.99 (0.93–1.05)	0.703
Completed Secondary School	0.93 (0.87–1.00)	0.067	0.96 (0.90–1.03)	0.296
Attended Tertiary Institution	1.00 (0.97–1.05)	0.800	1.03 (0.99–1.08)	0.145
**Occupation**				
Unemployed	Ref	Ref	Ref	Ref
Student	1.14 (1.04–1.26)	**0.006**	1.10 (1.00–1.20)	0.054
Formal employment	1.05 (0.96–1.14)	0.319	1.02 (0.93–1.11)	0.730
Informal Employment	1.07 (0.99–1.15)	0.086	1.05 (0.99–1.12)	0.057
Self-employed	1.06 (0.99–1.15)	0.090	1.05 (0.99–1.11)	0.086
**Monthly Household Income (KSh)** ^**b**^				
< 10,000	Ref	Ref		
10,000–50,000	1.03 (0.99–1.08)	0.152		
> 50,000	0.84 (0.56–1.24)	0.370		
**Key Population** ^**c**^				
No	Ref	Ref		
Yes	1.03 (0.93–1.14)	0.605		
**Number of HIVST Kits Offered**				
One Kit	Ref	Ref	Ref	Ref
Two Kits	0.92 (0.87–0.98)	**0.005**	0.93 (0.88–0.98)	**0.009**
**Ever Tested for HIV Before**				
No	Ref	Ref	Ref	Ref
Yes	0.95 (0.89–1.01)	0.124	0.96 (0.90–1.01)	0.109
**Current Testing Intent**				
Couple	Ref	Ref	Ref	Ref
Individual	1.46 (0.85–2.52)	0.168	1.49 (0.87–2.55)	0.147
**Sexual Relationship with Index Case** ^**d**^				
Relationship sex	Ref	Ref	Ref	Ref
Casual sex	0.92 (0.87–0.98)	**0.009**	0.93 (0.88–0.98)	**0.008**
Transactional sex	0.89 (0.84–0.94)	**<0.001**	0.90 (0.87–0.94)	**<0.001**
**HIV Risk Score Categorization** ^**e**^				
Low Risk	Ref	Ref		
Medium Risk	0.99 (0.92–1.07)	0.825		
High Risk	0.95 (0.84–1.07)	0.426		

^a^ Adjusting standard error for clustering at the 12 health facilities.

^b^ Average currency exchange rate over the data collection period: 1 USD = KSh. 110.60

^c^ Key population include PWID, AGYW, FF, FSW, MSM.

^d^ Coerced sex had only one respondent, hence not included in the regression.

^e^ Risk scoring based on sociodemographic and behavioral characteristics: Muttai et al, 2021.

### Qualitative analysis

Participants in the IDIs (N = 24) were closely balanced by gender, county, and HIV testing method selected (Table 3). About half of the participants were married and between ages 28 to 32 years.

**Table 3:** Characteristics of in-depth interview participants.

	Total (n = 24)
Characteristic	N (%)
**Gender**	
Male	11 (46%)
Female	13 (54%)
**Age**	
18–22 Years	3 (13%)
23–27 Years	5 (21%)
28–32 Years	11 (46%)
≥ 33 Years	5 (21%)
**Marital Status**	
Single	3 (13%)
In a relationship	6 (25%)
Married	12 (50%)
Widowed	1 (4%)
Separated	2 (8%)
**County**	
Homa Bay	13 (54%)
Kisumu	11 (46%)
**HIV Testing Method**	
Oral HIVST	12 (50%)
Provider-delivered Testing	12 (50%)

Using the theoretical framework of acceptability, the main emerging themes, related subthemes, and exemplar quotes are shown in Table 4.

**Table 4:** Themes and quotes from partner interviews.

Theme	Sub-theme	Exemplar Quote
Affective Attitude	Positive: HIVST are private / convenient	“…why I haven’t gone back to talk to her. And I told her that in the hospital where you called me there are a lot of people and they stare a lot…so I was scared. So she called me again and told me that there is something that I can use to test myself at home. So I told her that I am okay with that.”–[IDI, Female, APS+HIVST, Homa Bay]
	Positive: Provide Testing Choice	“The reason why I liked it and if I knew back then, I would have used it… I thought it was good because it was something that I would go with home and test myself. And he also told me that the results will just be the same as the one I would get from the hospital.”—[IDI, Female, Standard APS, Kisumu]
	Negative: Accuracy Concerns	“I think awareness is still low. Many people still don’t know about ST kit and those who know think it is not accurate. Myself, I did not know about the kit before I got the call…”—[IDI, Female, APS+HIVST, Homa Bay]
	Negative: HIVST in unknown	“I had never seen nor heard of the HIVST kit so I did not want what I have never heard of…I now hear about the HIVST, I would want to try that too although I’m still scared. Maybe they could counsel me first then introduce me to it”—[IDI, Male, Standard APS, Kisumu]
	Negative: Lack of testing support	“But my worry is if the result comes out positive and I’m alone in the house. Ooh sister, it is not easy. I think hospital is good because counselling will be done and also if one turns positive, they can be put into treatment and care right away.”–[IDI, Female, APS+HIVST, Homa Bay]
Burden	Positive: HIVST are convenient	“…you know most of the people don’t like time wastage, yes to come here and make queue, you know such things have discouraged people from coming here to the hospital but you know with that after you have acquired the kit you can do it at your own time.”–[IDI, Female, APS+HIVST, Homa Bay]
	Negative: Handling positive test result	“I prefer the standard test since I can be counselled and guided accordingly depending on the nature of the results. I’m not sure what I could have done had I turned positive when I tested myself in the house with oraquick or if we turned out to be discordant, what would the negative partner do to the other who is positive or vice versa.”—[IDI, Female, Standard APS, Kisumu]
Ethicality	Positive: HIVST promote confidentiality	“…picking them from the pharmacy really helps the youths because if you go there no one will know what they are going to pick from there… But at the facility from the time you enter the gate so many people know where VCT is so when they see you heading there they will start talking bad about you.”—[IDI, Female, APS+HIVST, Kisumu]
	Positive: HIVST promote privacy	“I liked it a lot. I was told to do the test in the morning… At that time my kids were still asleep. So I stayed privately in my bedroom and tested myself and privately saw my results. So I liked the privacy.”—[IDI, Female, APS+HIVST, Homa Bay]
	Positive: Promote couple testing	“…then there is also confidentiality, you know what you and your husband have tested there is a way that you just have confidence in that result or just the way that you have tested… this thing is good.”—[IDI, Female, APS+HIVST, Homa Bay]
	Negative: Sharing of partner’s contacts	“I would still want to know the person who gave my contact to sister. What stupidity is that? You know I have never mentioned any of my lovers anywhere. Why was he mentioning my name to sister?”–[IDI, Female, HIVST, Homa Bay]
Intervention Coherence	Negative: Mystery of testing through oral fluids	“Okay you know that process scared me a lot… I was like how can blood, if you remove the saliva around then you put it in that thing and you see something like blood coming out and I was like where has this blood come from and it was only saliva around my mouth.”—[IDI, Female, APS+HIVST, Homa Bay]
	Negative: Concerns about test accuracy	“The only question I had was the accuracy of that ora-quick, I was just worried it could bring positive results, yet I am negative, or it could even bring negative yet I am positive.”—[IDI, Female, APS+HIVST, Homa Bay]
Opportunity Cost	Negative: Transport cost to facilities	“…he asked me which pharmacy is close by where I stay… and that was the time I was going back to school so I just went back to school without picking the kits. Then they started calling me to go pick the kits but the challenge was the fare.”—[IDI, Female, APS+HIVST, Kisumu]
	Positive: Value for Money	“It is far because right now I would use 50 Kenyan shillings for transport… It can’t be affected because even if it is Long Distance and I want to go I will even walk. So that I can maintain the confidentiality and privacy. It is not very far.”—[IDI, Female, APS+HIVST, Kisumu]
Perceived Effectiveness	Positive: Reaching the unreachable	“I would recommend ST because many people fear injections and also, they can test in their houses privately. Many people are also busy with their work, with no time to come to the facility for tests. So, ST is still the best because they can do it anywhere at their own time.”–[IDI, Male, APS+HIVST, Kisumu]
	Positive: Reaching the interest group	“You know men fear coming to the hospital. They always fear that everyone in the health facility knows them and knows that they are unfaithful. So, many prefer self-test…”—[IDI, Male, Standard APS, Homa Bay]
	Positive: HIVST support couple testing	“When I got there, I asked for 2 ST kits then took one to my husband. My husband then tested himself first and that gave me a lot of courage. I looked at it and saw two lines and that really made me loose some breath. . . He then asked me to test myself too and the result was also positive… he tried to calm me down and told me to call sister so that we can go to [facility] for guidance on the way forward.”—[IDI, Female, HIVST, Homa Bay]
	Negative: Linkage to care concerns	“But with the blood test when I personally come here willingly like I have come they will test me and counsel me then put me on drugs. But with the other one someone will test themselves but maybe they are scared to come to the facility. So they will not come back…According to me I don’t think it’s good… Because with this the doctor will see the results… There is a way in which they talk to patients. And after that she will be put on drugs.”–[IDI, Female, Standard APS, Kisumu]
Self-Efficacy	Positive: Support from dispensers	“So they will just remove the self-test Kit because when you go there they must tell you that you are going to be given something like this. So they explain to you how you’re going to use it and what you’re going to do. You have to understand it first… So they teach you first and then they ask you if you know how to use it…”–[IDI, Female, APS+HIVST, Kisumu]
	Positive: HIVST are easy to use	“Like that one even if you have not gone to school you can just do it. Because it’s something that you just rub on your gums then you dip it there. The other one you have to measure the buffer. And when you put small amount or a lot the results might not be correct.”–[IDI, Female, APS+HIVST, Kisumu]
	Positive: Guidance from product inserts / instructions	“…they told me how they are used and inside there was a paper, and she was really explaining to me, so I was following the process and I ended up successfully testing myself by following the instruction as she had told me.”–[IDI, Female, APS+HIVST, Homa Bay]
	Negative: Low confidence in handling a positive test result	“But the blood test is better because the one for the gum you will be alone at home when doing the test. Your pressure will be high which is not good. But with the blood test you will be at the hospital and there will be counselors with you who will teach you everything and talk more about the virus with you.”–[IDI, Female, Standard APS, Kisumu]
	Negative: HIVST interpretation concerns	“The funny thing that I noticed with ora quick is that when you test and you exceed the time limit the results came out positive. So you must be explained to everything and be counseled so that you can know how to use it and use it well.”–[IDI, Female, APS+HIVST, Kisumu]
Contextual Factors	Facilitator: Staff influence	“Myself, I did not know about the kit before I got the call… You know, many people know that HIV lives in the blood and not the saliva…You know, when I went to pick it at the chemist, the guy explained to me many things about the kit, many people have used it and it is accurate so I trusted it. I think the guy empowered me a lot.”—[IDI, Female, APS+HIVST, Homa Bay]
	Facilitator: Local awareness efforts	“I heard it from the radio and also at the facility… Especially on the radio and here at the facility when you went you were told that if you want to test yourself and know your status you can take the self-test kit and go test yourself at home…They were just advising people to go for them at the pharmacy but they were still expensive at that time. When you are at the facility they give us for free.”–[IDI, Female, APS+HIVST, Kisumu]
	Facilitator: Staff follow-up	“… it was good since these people were doing the follow up… They were following whether I have gone to the chemist, taken the kit, tested myself and so on. Even after they counseled me they still kept on calling, so it was really good.”–[IDI, Female, APS+HIVST, Homa Bay]
	Facilitator: Supportive HIVST pickup locations	“No actually it was good; they didn’t ask me even a thing. I therefore perceived they were working together with these people. I am the one who asked how I would use the ora-quick and they explained everything to me. And it’s not like they will tell you about it where there are many people, she took me to a room at the back of the chemist and explained everything to me there.”–[IDI, Female, APS+HIVST, Homa Bay]
	Barriers: Losing out on other services at the facilities	“I would prefer the standard test since I sit down with the doctor for counselling, and this can help me a lot. You may also be informed about some new research information about HIV which you never heard of before. I can also be told of the way forward depending on the result e.g Test and treat.”—[IDI, Male, Standard APS, Kisumu]

While the framework provided perspectives from which to evaluate acceptability, and ensured all practical aspects of acceptability were evaluated, cross-cutting and logically linked sub-themes emerged within the thematic matrix (S1 Fig). Facilitation of HIVST utilization was mainly through inherent HIVST attributes, offering a testing choice which encompasses confidentiality and convenience. Barriers to HIVST utilization were mainly driven by factors around lack of awareness and limited provider support.

#### Affective attitude

We define affective attitude as how an individual feels about HIVST when offered within APS. Among the participants, there was very low awareness regarding the existence of HIVST as a testing option before participating in APS. In some cases, lack of awareness resulted in not having an opinion as to “whether it’s painful or how it feels like”, leading to preference for provider-delivered testing as familiar testing method. Largely those unaware who selected provider-delivered testing expressed curiosity to test using HIVST, with concerns relating to low confidence in handling a positive test result alone. Specific rumors about cases where “people who turned positive end up committing suicide” while testing away from the health facility heightened their fears. On the other hand, the participants acknowledged that HIVST came with many benefits, including easily knowing the status of a new sexual partner, no injections and pricks as compared to the provider-delivered testing, in addition to the ease of collecting the test kits at designated community pharmacies. The privacy and convenience that comes with testing from the comfort of their home gave HIVST some additional preference.

Participants also felt that HIVST provided testing options, especially for “men who fear coming to the hospital”, “those who fear injections”, “people who don’t like time wastage” relating to waiting times and repeat counselling at the health facilities, “those busy with work and studies” and “people who don’t like to share their results”.

#### Burden

We define burden as perceived amount of effort required to test using HIVST within APS. Resulting from inherent fear of injections, some partners considered HIVST as needle-free hence preferred because there was no burden of pain. In addition, those with a busy schedule were relieved of the burden of visiting the health facility, hence HIVST was preferred. While those originally aware of HIVST were confident and expressed the need to reach out to the health facility for positive test confirmation, to others, handling a positive test result especially where the couple is discordant was a scary process to undergo alone without proper counseling support. In which case provider-delivered testing was preferred because that burden will then shift to the provider to ensure the results are accepted by both parties and avoid potential partner violence.

#### Ethicality

We define ethicality as the extent to which HIVST, within APS, was a good fit with an individual’s value system. Participants considered HIVST as confidential and private, starting from the collection of HIVST kits at the community pharmacy where no one can easily tell the purpose of the visit and in some pharmacies invitation to “a room at the back”, to the private testing within closed spaces in the household, and finally to confidential results known only to the person concerned without fear of the results being “told to other people”; a fear associated with provider-delivered testing. To others, presence of HIVST in the household is likely to result in discomfort once family members see it especially if they were not involved. Female participants reported that HIVST promoted couple testing, especially in cases where the spouse does not usually go to the health facility. Couple testing was also deemed to promote disclosure, in cases when one has already received a positive test result at the health facility.

Some participants felt that the process of identifying sexual partners through partner notification was a violation of their privacy. The idea of sharing sexual partner’s contact without their knowledge agitated some participants, although they also perceived sharing their own sexual contacts as helping those who have been exposed to also know their status.

#### Intervention coherence

We define intervention coherence as the extent to which the individual understands HIVST within APS and how it works. With little awareness of HIVST among participants before the intervention, testing using oral fluids presented as a mystery. The conviction that HIV “lives in the blood and not in the saliva” made the participants doubt the accuracy of HIVST. With limited knowledge, some participants relied on assurance by HTS providers and pharmacy dispensers on accuracy and reliability of HIVST. Concerns about accuracy persisted even among those who were aware of HIVST, resulting from need for confirmatory testing and specific reading time of test results.

#### Opportunity cos

We define opportunity cost as the extent to which benefits, profits, or values must be given up to engage in HIVST within APS. Whereas HIVST kits were provided free of charge within the study, where participants had to travel to designated community pharmacies, they reported spending between Kenya Shilling (KSh) 30 to 100 on transport (~0.26–0.87 US dollars at time of data collection). One participant indicated travelling three kilometers while another reported travelling 20 minutes on a motorcycle. While most participants were conveniently close to the pharmacy, for some the transport cost was prohibitive and forced them to make compromises on some other need. Despite access to the pharmacy being a potential barrier for those who needed transport, one respondent shared that there was value for money in assured privacy and confidentiality by testing using HIVST.

#### Perceived effectiveness

We define perceived effectiveness as the extent to which HIVST is perceived as likely to contribute to the objectives of APS. The participants reported that HIVST could help reach certain population segments within the community who may not routinely be available for provider-delivered testing within APS, including those with “fear” of loss of confidentiality, public stigma, or perhaps self-stigma, from testing at health facility HIV testing units. This stigma emanates from “people talking badly” about them, healthcare workers (HCWs) who know them or people known to them who might think they are “promiscuous”, fear of positive status not being kept a secret and test results being announced to the public. Additionally, those with a busy lifestyle who could not physically attend health facilities were identified as potential beneficiaries of HIVST due to flexibility offered in timing and location testing. A few participants expressed concerns about “time wastage” at the health facility, mainly attributed to repeat counselling sessions for frequent testers or long queues to seek services.

To others, HIVST provided an option to effect couple testing, and even to test casual and stable sexual partners. It provided an avenue to effect disclosure, to engage their partners and build interest in their health status and health seeking behavior. A few participants expressed concerns with processes after testing at home using HIVST, and whether those tested would be willing to come to the health facility easily should they get a positive test result. However, among those who were already aware of HIVST, they reported awareness of the need to access the health facility for test confirmation following a positive test result.

#### Self-efficacy

We define self-efficacy as the participant’s confidence that they can complete HIVST within APS. While participants who were already aware of HIVST shared that it was easy to use by simply “rubbing the gum”, others found it useful that instructions were shared by the dispensing and study staff, while others found it beneficial to refer to the product inserts or call the counselors for guidance. A few participants who opted for standard APS, shared concerns about inability to read the test kit, and were further concerned about possible “gaps” in providing the instructions. Other participants shared concerns about the test accuracy, and effect of reading time on test outcome. Participants also shared concerns about low self-efficacy in handling a positive test result, and doubted whether they would be able to handle it “alone” without counselling and support from a HCW.

#### Contextual factors

We evaluated contextual factors that serve to promote or hinder HIVST implementation within APS, presenting as logistic or health system factors that may have direct or indirect impact on the experience of the participants.

*HIVST facilitators*. Few participants were aware of HIVST before rollout within APS, acquiring that knowledge and experience from the media and prior testing interaction with HIV testing providers. A few shared that they used HIVST after their fears and concerns were addressed by the HTS providers. While HIVST was promoted within APS, many partners related its application to risk-based testing, with some pointing out the ability to “test new sexual partners before sex”. While some participants expressed concerns with “being alone” during testing, others highlighted the remote support received from HIV testing providers all through from deciding to test, collecting the test kits, the testing process and dealing with the test results. They expressed a high level of satisfaction resulting from feeling “empowered”, how the HTS provider “talked well” to them encouraged openness, drawing them in to feel “close and comfortable” hence building strong relationships that facilitated testing and linkage to care. Most participants were comfortable with the procedures at the pharmacy and perceived it as discrete and well-coordinated by the APS staff.

*HIVST barriers*. While HIVST was provided free within APS, participants expressed difficulties in accessing additional kits outside of the study due to cost and uncertainty of where to get them. Participants appreciated confidentiality that manifested when unique alpha-numeric codes were sent to their phones, which were used to pick up HIVST at pharmacies. A few described the text message as confusing, as they were not able to decipher the meaning. As other participant pointed out concerns associated with provider-delivered testing, a few pointed out to what they are losing out on by not going to the health facilities including access to other HIV prevention information and professional handling especially for couples.

## Discussion

In this study evaluating HIVST acceptability among partners receiving APS, we found high uptake of HIVST (82%) and high acceptability. We determined lower HIVST preference among those with high-risk sexual exposures involving transactional or casual encounters. In this context, ‘Intervention coherence’, ‘self-efficacy’ and ‘ethicality’ presented as the three key theoretical framework of acceptability constructs, mediated by factors relating to testing convenience, confidentiality, provider support and HIVST awareness. HCWs play a critical role in creating HIVST awareness and providing acceptable solutions to fears around couple testing and handling positive test outcomes. This suggests that APS implementation should focus on empowering the partners to make HIV testing decisions fit for their individual scenarios.

HIVST uptake rates in this study are within the range of those found in the literature of 22 to 87% [17], suggesting that acceptability varies across populations. High preference for HIVST has been demonstrated among those with transactional sex encounters in Zambia (93%) [24], 98% and 82% among pre-exposure prophylaxis (PrEP) users in Kenya [38] and Uganda [39] respectively. The high uptake resulted from positive attributes of HIVST including confidentiality and convenience, ease of use and circumventing fears concerning testing at the health facility reported in the qualitative interviews, while the low awareness may have been due to a large number of participants from the rural region of Homa Bay County. In addition, high uptake of HIVST may be a result of reported close relationship between clients and APS HTS providers [40], which is necessary for successful implementation of APS [7], and further captured in the qualitative findings. Our findings are in line with a study in South Africa that showed low awareness level did not influence HIVST uptake [41], signifying impact of educating the partners and their level of trust in HCWs.

We found that HIVST use was acceptable across socio-demographic characteristics, similar to a study in Kenya which found HIVST use among healthcare workers was not associated with age, previous HIV testing or sexual behavior [20], but was associated with being female which was different from our findings. A population based survey in Kenya showed higher prior HIVST utilization among those in high HIV prevalence areas, higher education level, higher health quintiles, higher number of sexual partners and lower age categories [42]. Different from our study, this study evaluated uptake based on prior utilization and not revealed preference. Key factor reported in our qualitative interviews, is the direct influence of the HTS providers in the decision process, which may have made it difficult for our analysis to discern socio-demographic influence.

A novel finding from our study is that HIVST use is lower among those partners who engaged in casual or transactional sex with an index client, compared to sexual encounters within relationships. While few participants reported being single, more reported casual or transactional encounters alongside their main relationship. From qualitative data, participants expressed concerns with HIVST kits being seen within the home, and potential of relationship conflict if the partner was not involved. This may explain preference for provider-administered testing for those whose encounter was outside the relationship and identify as being married or in a monogamous relationship. Awareness and accuracy concerns with HIVST were expressed in the qualitative interviews and may also have been a factor contributing to provider-delivered testing preference for those who perceived themselves to be at increased risk due to transactional or casual sexual encounter. In the STAR demonstration project in SSA, high-risk individuals self-selected for provider-administered tests citing higher sensitivity as important due to their high-risk exposure [43]. Similarly in a study in Malawi, participants who did not choose HIVST were more likely to worry a lot about having HIV [44]. Given APS has been shown to be particularly acceptable for identifying casual and non-primary partners for those with multiple partners [45], HIV testing options for the identified partner should take into consideration the testing preference considering the need to preserve their main relationship.

Quantitative data show that HIVST use was less likely among those offered an additional kit compared to those offered one kit. This comparison was across different time periods, and different study populations, hence findings are likely to be affected by temporal trends or implementation factors. Further research may be needed to discern the effect of an additional test kit on acceptability and uptake of HIVST. Qualitative data however highlight fears associated with navigating couple testing, including discordant results and potential for intimate partner violence. While a large majority of our participants reported being monogamously married or in a cohabiting relationship, almost all participants had the intention of testing as an individual. This being a group already informed of a possible HIV exposure, in some cases the need to know their status on their own took precedence over couple testing as reported in qualitative interviews. Sensitization on HIVST should emphasize the risks associated with use of HIVST as a screening tool for potential partners, and the required support for couple testing.

The theoretical framework of acceptability defines acceptability as a multi-faceted construct [36], recognizing that not all acceptability constructs may be relevant to the intervention [46]. ‘Perceived effectiveness’ focused on the ability of HIVST to reach new populations. Despite being a significant decision-making factor at the individual level, and would largely address client options under APS, it remains a factor least likely to be altered by the provider. Identification of those who fear health facilities, don’t have time to wait at a health facility, fear pricks or have strong preference for confidentiality should be encouraged during APS implementation, to enable suggestion of suitable testing alternatives including HIVST. The feelings under ‘affective attitude’ were largely informed by how participants perceived the other acceptability constructs, while under ‘opportunity cost’ we identified transport cost as the main concern, which is a limitation of interventions not being offered door-to-door. ‘Burden’ presented in terms of busy personal schedule and handling a positive test outcome, with the former relating to ‘perceived effectiveness’ and the latter relating to ‘self-efficacy’. In this intervention combination and context, ‘intervention coherence’, ‘self-efficacy’ and ‘ethicality’ presented as the three key acceptability constructs, influenced by key factors such as testing convenience, confidentiality, provider support and HIVST awareness. Low awareness, coupled with lack of understanding of oral fluid antibody testing, may have led to concerns around accuracy hence significance of ‘intervention coherence’. This underscores the importance of sensitization and education of the target population to increase acceptability. Low awareness rate may also have resulted in reduced self-efficacy in conducting self-test and ability to handle a positive test result. While ‘ethicality’ remains the least measured construct in published theoretical framework of acceptability application [46], it manifested in terms of confidentiality, convenience, privacy, couple testing and disclosure, and was significant in influencing HIVST acceptability.

Our study had several limitations. First, the qualitative interviews were conducted within the health facilities. Response bias may have played a role where partners responded in a manner to please the interviewer. To minimize this, external non-study interviewers conducted the interviews and were trained on the interview guide to elicit unbiased responses from interviewees. Second, the study provided option for one or two HIVST kits in a sequential manner, hence differences in population or temporal trends may have affected the observed outcome. While not the best design to study effect of an additional kit, we used this variable to control for effect on testing method selected. Third, our findings may have limited generalizability as intervention acceptability vary by geography and culture [46]. We, however, ensured balanced demographic representation. Fourth, the parent study provided oral fluid-based HIVST, hence limiting interpretation of our findings to oral fluid-based tests kits. Future studies could investigate acceptability and related factors when blood-based HIVST are provided as an option. Finally, using existing models to synthesize qualitative interviews may limit presentation of certain contextual factors. Relatedly, our interview guide did not directly match the theoretical framework of acceptability construct but was broad enough to elicit aspects covering all the constructs and beyond. The framework has been successfully applied in different ways, including `partially, during analysis and presentation [40, 46], resulting in informative findings.

We find high uptake and acceptability of HIVST within APS regardless of partner demographics. Offering HIVST has potential to increase testing uptake and may improve efficiency of the health system in implementing APS. More awareness and provider support may be needed to increase HIVST uptake among those with casual or transactional APS sexual encounters. Providers should consider context of the partner’s sexual encounter and extend counselling support when recommending HIVST within APS.

## Supporting information

S1 Fig Thematic matrix of HIVST acceptability within APS, applying the theoretical framework of acceptability by Sekhon et al. (TIF)
