# Supplementary figures and images for: Uptake and acceptability of oral HIV self-testing in the context of assisted partner services in Western Kenya: A mixed-methods analysis
Source: PLOS Glob Public Health. 2024 Nov 15;4(11):e0003960. doi: 10.1371/journal.pgph.0003960 (PMC11567626; doi:10.1371/journal.pgph.0003960)

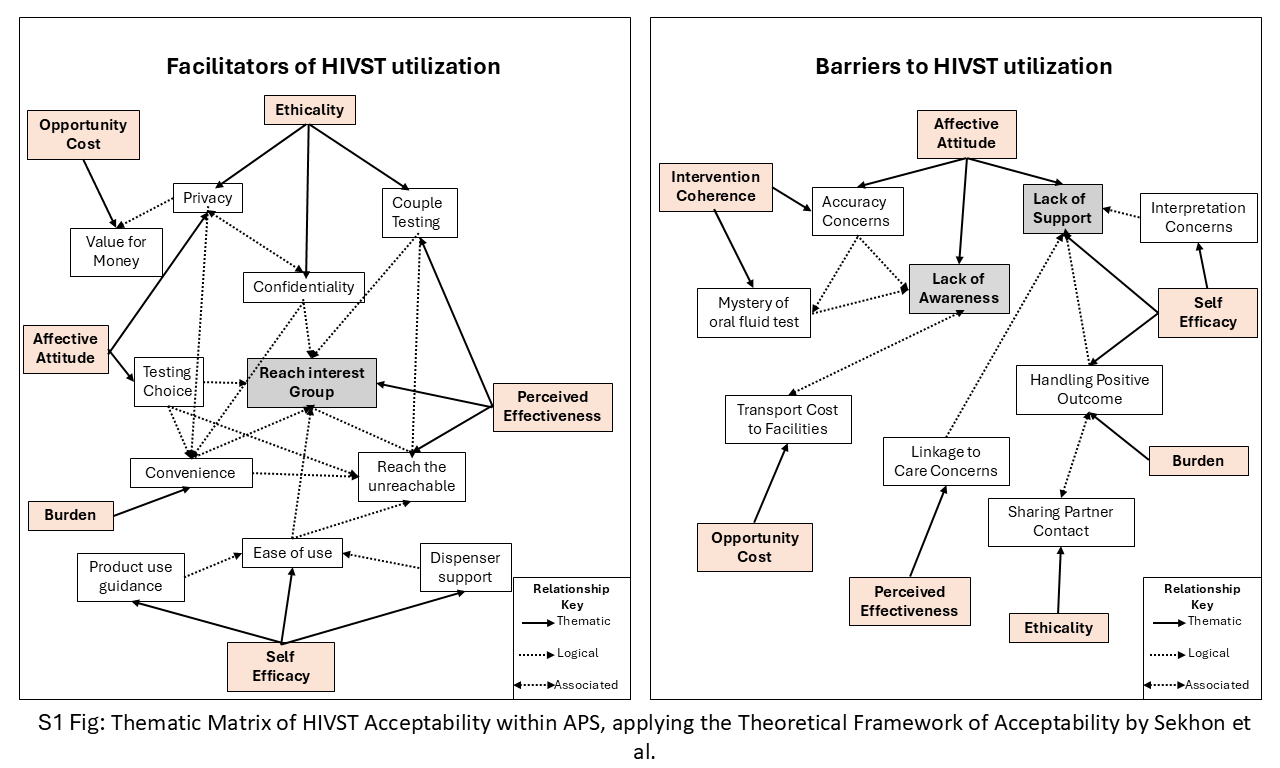

Supplement: S1 Fig — (TIF) [file pgph.0003960.s001.tif]
